# Sulfonated chitosan interlayer derived from marine crustacean waste for overcoming the permeability–selectivity trade-off in polyamide desalination membranes

**DOI:** 10.1039/d6ra04009h

**Published:** 2026-07-03

**Authors:** Fahad Ayesh Alharthi, Hamed M. Al-Saidi, Salman. S. Alharthi

**Affiliations:** a Water Technologies Innovation Institute & Research Advancement (WTIIRA), Saudi Water Authority (SWA) PO Box 8284 Al-Jubail 31951 Kingdom of Saudi Arabia FAlharthi3@swcc.gov.sa; b Department of Chemistry, University College in Al-Jamoum, Umm Al-Qura University Makkah 21955 Kingdom of Saudi Arabia hmsaidi@uqu.edu.sa; c Department of Chemistry, Taif University, College of Science P.O. Box 11099 Taif 21944 Kingdom of Saudi Arabia

## Abstract

Annually, around 6–12 million tons of marine crustacean waste are generated by the seafood industry, representing an abundant and underutilized resource. Valorization of this waste into high-value functional materials provides both environmental and industrial benefits. On the other hand, overcoming the trade-off between water permeability and salt rejection in polyamide (PA) membranes remains a critical challenge in desalination. Therefore, this work presents a novel strategy for fabricating high-performance thin-film composite polyamide membranes by employing a sulfonated chitosan (SC) interlayer derived from marine crustacean waste, offering a sustainable approach to overcome the inherent permeability–selectivity trade-off in desalination membranes. Chitin extracted from marine crustacean waste was converted into chitosan *via N*-deacetylation and subsequently functionalized into SC. The obtained SC was employed as a hydrophilic and negatively charged interlayer to fabricate a thin-film composite membrane. Firstly, a polyacrylonitrile (PAN) substrate was prepared *via* phase inversion, followed by deposition of the SC interlayer through ultrafiltration. The PA layer was then formed *via* interfacial polymerization (IP) to obtain a composite membrane containing SC interlayer (PAN–SC–PA). Comprehensive structural and morphological studies using various characterization techniques confirmed that SC exhibits reduced crystallinity and a nanoporous structure compared to pure chitosan, that facilitates improved interfacial compatibility and controlled monomer diffusion during IP. The incorporation of the SC interlayer significantly enhanced membrane performance, where the optimized PAN–SC–PA membrane showed a remarkable water permeance of 25.6 L m^−2^ h^−1^ bar^−1^, corresponding to an increase of approximately 1064% compared to the control PAN–PA membrane, while maintaining an excellent Na_2_SO_4_ rejection of 99.02%. The significant enhancement in membrane performance is attributed to the synergistic effects of improved hydrophilicity, surface charge, and enhanced interfacial structure due to the presence of the SC interlayer. The results of this study highlight the waste-derived functional interlayers as an effective strategy to overcome the permeability–selectivity trade-off in advanced desalination membranes.

## Abstract 2

This work presents a novel strategy for fabricating high-performance thin-film composite polyamide membranes by using a sulfonated chitosan interlayer derived from marine crustacean waste, offering a sustainable approach for desalination membranes.

## Graphical abstract

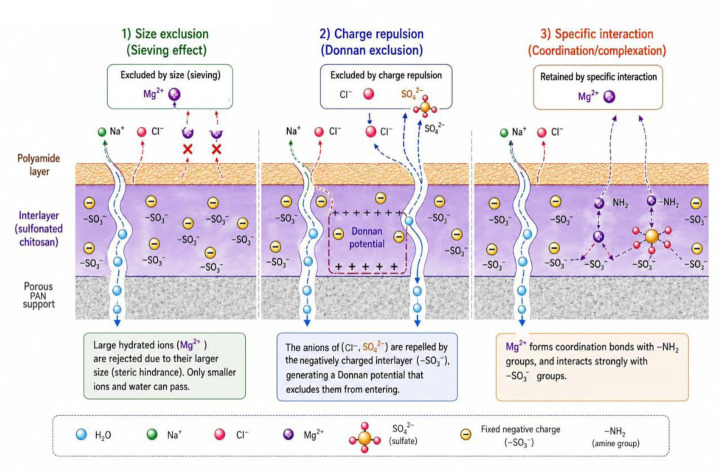

## 1. Introduction

Nanofiltration (NF), a filtration process using nanoporous membranes, plays an important role in water purification and desalination. NF usually uses a pressure-controlled membrane where this technique can separate a variety of ions with different valences and many organic compounds at relatively low operating pressures.^1^ Most commercial PA membranes are prepared by IP where a PA layer is formed as a result of the reaction of two monomers at an interface between two immiscible solvents. The two monomers are usually a multifunctional amine dissolved in water and acyl chloride prepared in organic solvent.^2^

Among different preparation techniques, IP is useful in controlling the polymerization reaction and therefore improvement of the properties of the prepared membranes. However, some polyamide membranes prepared by IP suffer from low filtration efficiency because of the preference influence between selectivity and permeability *i.e.* the permeability–selectivity trade-off. This effect discusses the inherent inverse relationship where increasing water permeability leads to a decrease in solute rejection.^3^ PA membranes display a moderate hydrophilicity because of the presence of some polar functional groups *e.g.* amide and carboxyl moieties. However, this intrinsic property is often insufficient to achieve advanced desalination performance, particularly in overcoming the permeability–selectivity trade-off and mitigating fouling. Thus, a lot of research effort has been made to improve PA membranes efficiency.^4,5^ Two-dimensional materials were used in an attempt to break trade-off effect. The idea of this research is based upon the use of vermiculite modified by cobalt.^4^ However, the permeability of water was slightly improved (more than ≈100). Solvent activation was employed to improve the conditions of IP reaction where a mixture of dimethyl sulfoxide and water worked effectively to active PA membranes used in reverse osmosis.^6^ Although the use of highly toxic solvent (DMSO), the enhancement rate in water permeability is very low (≈43%). A multifunctional aqueous phase was recently designed as a modifier for IP where an ammonium monomer was prepared and used in IP process.^7^ The incorporation of this monomer enhanced the crosslinking providing pore structures which enhances water flux. Recently, an effective methodology has been developed to overcome trade-off effect, and therefore, improve the efficiency of polyamide membranes. The methodology is based upon introducing an interlayer between PA layer and substrate.^8–12^ The presence of interlayer modulates substrate properties making the interaction between substrate and monomers used in interfacial polymerization better *via* homogeneous distribution of multifunctional amine solution during IP process.^13^ In other words, the interlayer in polyamide membranes services as a functional bridge that regulates IP, improves adhesion of layers, reduces defects, and tailors surface charge thereby simultaneously increasing water flux and salt rejection.^14^ Thus, it can be said that introducing an interlayer into PA membranes provides an ideal solution to the trade-off effect.^10^ Numerous organic materials containing active functional groups, including polydopamine, polyethyleneimine, tannic acid, and polysaccharides like pure chitosan, have been widely employed as interlayers to regulate interfacial polymerization and enhance membranes performance.^15–17^ However, some of these membranes still suffer from low membrane efficiency in terms of water permeability and/or rejection rate. Despite the notable progress in membranes engineering, there remains a pressing need to test a broader spectrum of organic materials, both naturally derived and synthetically engineered, as interlayers. Such efforts are essential to further optimize membrane structure–performance relationships and to overcome existing limitations in separation efficiency.

Chitosan, composed of glucosamine units and acetyl glucosamine units, has attracted considerable attention as a membrane material due to its biodegradability, hydrophilicity, and the presence of active functional groups such as amino (–NH_2_) and hydroxyl (–OH) moieties.^18^ Globally, an estimated 6–12 million tons of marine crustacean's waste are produced annually. The chitin content in this crustacean's waste is approximately 15–40%.^18^ Therefore, chitosan derived from chitin isolated from marine wastes can be considered a cost-effective and economically viable material in membranes engineering. However, the use of pure chitosan in membrane-based separation processes remains limited due to several intrinsic drawbacks. First, pure chitosan exhibits weak mechanical strength and structural integrity, particularly under hydrated conditions, which restricts its long-term operational stability.^19^ Second, the high swelling tendency of non-modified chitosan in acidic aqueous environments leads to significant changes in pore structure. This reduces selectivity and membrane performance.^20^ Additionally, pure chitosan-based membranes suffer from low chemical stability, since protonation of amino groups under acidic conditions can result in partial dissolution or structural deformation.^21^ Finally, the lack of a well-defined and tightly controlled nanostructure limits chitosan ability to achieve high salt rejection. On the other hand, most previously reported chitosan-based interlayers mainly act as intermediate layers to regulate monomer diffusion and improve interfacial compatibility. In contrast, chitosan derivates containing active functional groups not only regulates interfacial polymerization but also introduces effective groups that provide a stable negative surface charge over a wide pH range, thereby enhancing Donnan exclusion and contributing to improved slats rejection.^3^ Consequently, various modification strategies of pure chitosan, such as crosslinking and functionalization, have been explored to enhance its mechanical stability, reduce swelling, and therefore improve separation efficiency. The functional groups available in chitosan, especially the hydroxide groups, make the chemical functionalization easily. Sulfonation of pristine chitosan meaningfully enhances the physicochemical properties of chitosan by increasing its hydrophilicity, surface negative charge density, pH stability, and compatibility with polyamide formation, thereby improving membrane permeability, and selectivity.^16^ Although sulfonated chitosan provides attractive features in the field of membranes engineering, to the best of our knowledge, systematic studies on the employee of sulfonated chitosan derived from marine crustacean waste as a functional interlayer for thin-film composite polyamide membranes remain very scarce.

The present study aims to valorize marine crustacean waste by converting it into sulfonated chitosan, which is subsequently employed as an interlayer in polyamide membranes. This approach is designed to mitigate the inherent trade-off effect between permeability and selectivity, thereby significantly enhancing desalination performance of PA membranes. The composition, morphology and the surface properties of all prepared membranes will be characterized. Moreover, the performance of membranes will be investigated and discussed in detail.

## 2. Experimental materials and methods

### 2.1. Instrumentations

ATR-FTIR spectra of materials under investigation were recorded by JASCO FT-IR spectrometer (model 4600) in spectral high resolution of 0.7 cm^−1^ and the range of 400–4000 cm^−1^ (Tokyo, Japan). The crystal type of IR spectrometer is monolithic diamond crystal. A JEOL JEM-6390 scanning electron microscope combined with energy dispersive X-ray spectroscopy was employed to study morphology and elemental distributions of isolated chitosan, SC, and prepared membranes. Spectrophotometric measurements were carried out using Shimadzu UV-visible spectrophotometer (model UV-1280, Japan). Atomic force microscopy with XY scan range of 90 × 90 µm, while, *Z*-range is 7 µm (Dimension Icon, BRUKER, USA) was used to study the roughness of the membrane surface. The zeta potential of the membrane surface was analyzed by surface zeta potential meter (SURPASS-3, Anton Paar, Austria) with 0.001 mol L^−1^ KCl aqueous solution. A contact angle measuring instrument (DSA25, KRUSS, Germany) was employed to measure the water contact angle (WCA) of the membrane at 25 °C. The volume of water drop used for measurement was 3 µL. The standard porosimetry method was applied to measure the porosity of the developed membranes by Porosimeter model 3.1 instrument (Porotech Ltd, Ottawa, Canada) in the presence of *n*-octane as a reference liquid. Conductivity meter from HANNA (Rhode Island, USA) was employed to measure the salt concentration. The concentrations of ions were determined by ion chromatograph model Dionex™ Inuvion™ IC System from Thermo Fisher (Waltham, Massachusetts. USA).

### 2.2. Materials and reagents

All chemicals and solvents applied in this work were highly pure and did not require prior purification. Shrimp used to isolate chitin was collected from fish market in Taif, Saudi Arabia. Polyacrylonitrile (PAN, *M*_w_ = 80 000 Da) was obtained from Sigma-Aldrich (Saint-Louis, Missouri, USA). Commercial polyethylene terephthalate (PET) sheet with the thickness of ≈90 µm was cut in the size of 4 × 4 cm and used as a support for PAN thin film. Trimesoyl chloride (TMC, 98%), piperazine (PIP, 98%), chlorosulfonic acid and *n*-hexane (97%), were obtained from Sigma-Aldrich (Darmstadt, Germany). The salts, acids, and bases used in this work were purchased from Sigma-Aldrich (Darmstadt, Germany). Ethylenediamine (ED) and polyethylene glycol (PEG) with molecular weight = 1000 Da were obtained from Sigma-Aldrich (Darmstadt, Germany).

### 2.3. Isolation of chitin from Shrimp shells and its conversion into chitosan

The method previously mentioned in our work^22^ was followed for isolation of chitin and its conversion into chitosan. Briefly, the fine powder obtained from Shrimp shells was treated by diluted HCl for 2 h at 25 °C, followed by washing using suitable amount of deionized water and drying for 24 h at 60 °C. NaOH (2 mol L^−1^) was used for deproteinization where the powder was refluxed for 19 h. Color removal was performed using a mild oxidizing treatment (KMnO_4_ + oxalic acid + H_2_SO_4_). The sample was washed by hot ethanol and boiled in acetone to remove the impurities. The chitosan was obtained from chitin by refluxing with 50% NaOH for 18 h. The extracted chitosan was washed by hot distilled water to neutrality. Chitosan was dried at 50 °C for 24 h. For purification, the chitosan was dissolved in 4% acetic acid. The chitosan was characterized using SEM-EDX and XRD.

### 2.4. Preparation of sulfonated chitosan

SC was prepared by the reaction between chitosan and chlorosulfonic acid (Sigma-Aldrich, Darmstadt, Germany) according to the method previously mentioned in ref. 23. 100 mL of dichloroethane was added to 5 g of chitosan with continuous stirring for one hour at 25 °C. An appropriate amount of chlorosulfonic acid (0.030 mol) was added gradually with stirring in an ice-bath at 3 °C for 30 min. The SC was then removed from the mixture by filtration, washed by dichloroethane, and finally dried at room temperature.

### 2.5. Preparation of PAN substrate

The phase inversion method was used to prepare PAN substrate according to the following procedure: a mixture of PAN powder (12 w/w%) and PEG-1000 (3 w/v%) was dissolved in DMF with stirring strongly at 70 °C for 10 h to get casting solution. To remove gas, the previous solution was left for 36 h. Then, the casting solution was spread on polyethylene terephthalate (PET) sheets fixed on glass plates by a custom-made Doctor's blade (100 µm slit width). Immediately after casting PAN solution, substrates were dipped in deionized water and allowed to stand, leading to phase inversion. Hence, substrates were fabricated.

### 2.6. Formation of SC interlayer on PAN substrate (PAN–SC membrane)

Dead-end ultrafiltration was employed for deposition of SC interlayer on porous PAN substrate using an ultrafiltration cell at 3 bars. An aqueous SC solution of 1 w/v% was prepared by dissolving in 4% v/v acetic acid with stirring in an ultrasonic bath for 30 min at 25 kHz. Then, aqueous SC solution was filtrated through porous PAN substrate. After the end of the filtration process, the former membrane was stored in deionized water. The formation of SC interlayer was confirmed by FT-IR spectrum and EDX measurements.

### 2.7. Fabrication of PAN–SC–PA membrane

IP technique was applied for the PA layer formation on the surface of PAN–SC membrane. For IP, PAN–SC membrane fixed inside of a laboratory-made frame was firstly dipped in aqueous PIP solution (0.1 wt%) for 3 min. Then, the organic TMC solution (0.05 wt%) was poured on the PAN–SC for 1.00 min. The formed PAN–SC–PA was then washed using *n*-hexane to remove the excess of TMC. Lastly, the PAN–SC–PA membrane was dried for 20 min at 60 °C. For comparison, the same IP conditions was employed to fabricate the PAN–PA membrane.

### 2.8. Evaluation of membranes efficiency

**Fig. 1:**
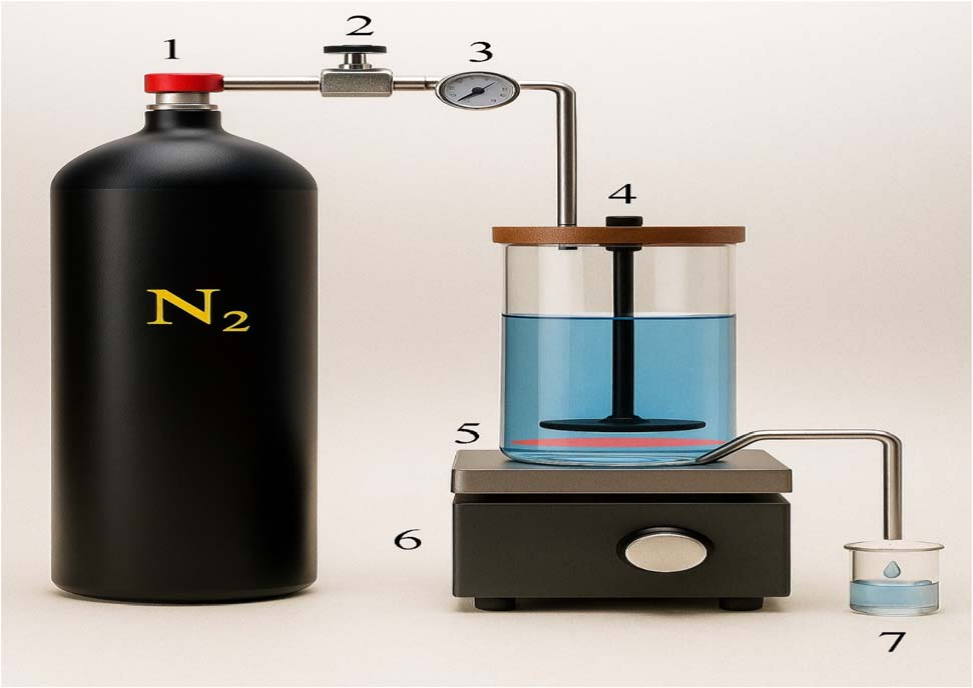
Schematic diagram of stirred ultrafiltration unit designed in our laboratory. (1) nitrogen cylinder, (2) regulator, (3) manometer, (4) stirred ultrafiltration cell, (5) a membrane under investigation, (6) magnetic stirrer, (7) water container.

The membranes performance was tested using a stirred ultrafiltration cell designed in our laboratory as shown in Fig. 1. The unit is similar to the one used in ref. 24 with some modification. The measurements were carried out at 25 °C, whereas, the stirring speed ranged from 250 to 300 rpm. To measure water flux, pure water was filtrated at a pressure in the range of 1–5 bar for 25 min. After obtaining the stationary ultrafiltration mode of the membrane, water flux (*J*) in the unit of L m^−2^ h^−1^ was determined using eqn (1):1
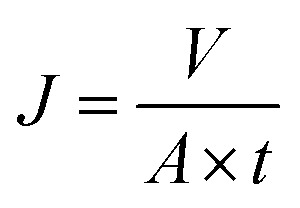
where, *A* is the membrane area in m^2^, *V* is the water volume in liter passing through the membrane, while, *t* is the time of filtration in h.

The water permeance (*P*) in the unit of L m^−2^ h^−1^ bar^−1^ can be calculated by eqn (2):2
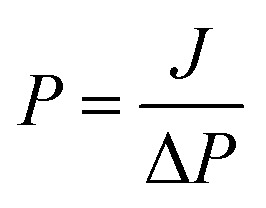
where, Δ*P* is applied pressure difference in bar.

The rejection ratio of salts (*R*, %) is calculated using eqn (3):3
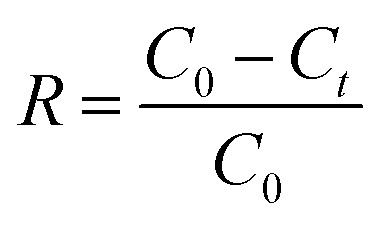
where, *C*_0_ is solute concentration in feed solution, while, *C*_*t*_ is the concentration of solute in permeate solution. The concentrations were measured in g L^−1^ and determined by conductivity meter in the case of salts and an ion chromatograph in the case of ion.

The selectivity of prepared membranes (*α*), also known as separation factor, for either ions or salts was calculated using eqn (4):4
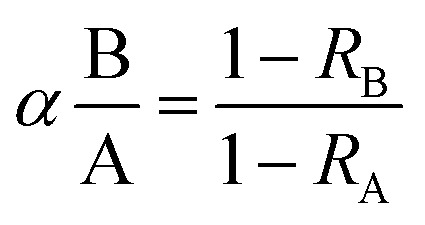
where, *R*_B_ and *R*_A_ are the rejection rates to B and A, respectively.

## 3. Results and discussion

We have already isolated chitosan from Shrimp shells and studied the chemical structure and morphology in detail in our work.^22^ However, the purity of chitosan extracted in this work was tested by ATR-FTIR spectrum. The most characteristic frequencies are at 3450 and 1658 cm^−1^ corresponding to NH_2_ group and CO stretching-amide I, respectively. Moreover, the degree of deacetylation (DDA) of chitosan was estimated by ATR-FTIR spectrum with the aid of equation mentioned in ref. 19 to be 93%. This value indicates that 93% of *N*-acetylated groups available in chitin have been converted into NH_2_ groups. Therefore, it is expected that the chemical modification will be easily due to the presence of NH_2_ groups. On the other hand, the crystallinity index (CI) was calculated by the X-ray diffractogram according to the equation mentioned in our work.^22^ CI was 86.3% in agreement with the result previously obtained in ref. 22. Therefore, highly crystalline chitosan is not ideal for the manufacture of PA membranes because of its dense chain packing, limited functional group accessibility, and restricted mass transport, which collectively hinder interfacial polymerization and reduce water permeability.^25^ Hence, the chemical modification of this material becomes necessary.

### 3.1. Characterization of SC

**Fig. 2:**
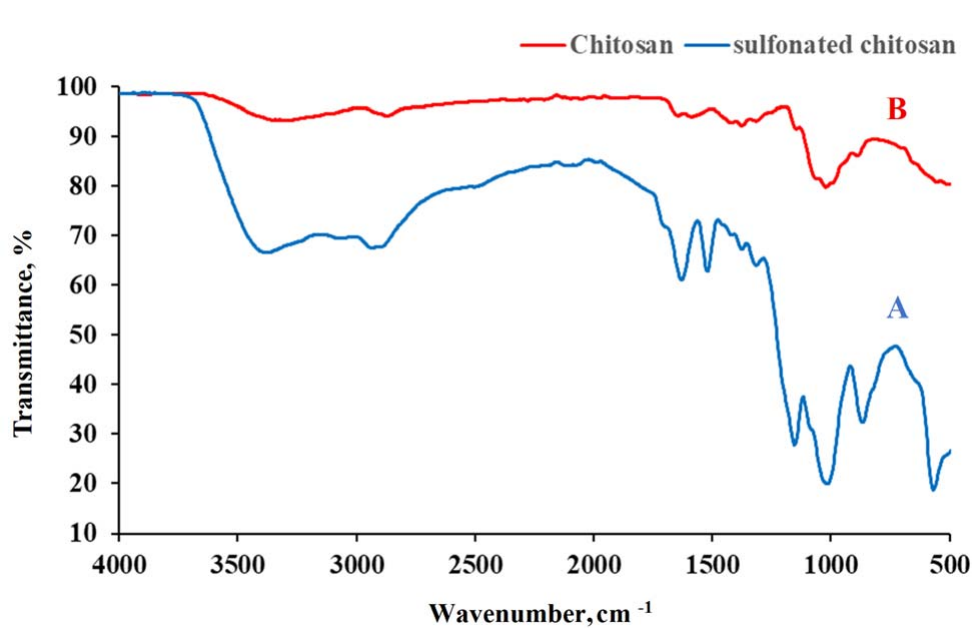
The ATR-FTIR spectra of (A) SC, (B) non-modified chitosan.

The chitosan sulfonation was established by observation of characteristic peaks in ATR-FTIR spectrum of SC shown in Fig. 2A at 1034 and 1192 cm^−1^ with the enhancement of broad band intensity at 3220 cm^−1^ compared to the spectrum of pure chitosan shown in Fig. 2B. These characteristic peaks correspond to stretching vibrations of O=S=O group where the band at 1034 cm^−1^ reverts to symmetrical vibrations, while, the one at 1192 cm^−1^ is due to asymmetric vibrations. The enhanced intensity of broad band at 3220 cm^−1^ after sulfonation of chitosan can be attributed to the introduction of highly polar –SO_3_H groups, which strengthen hydrogen bonding, alter the local electronic environment, and reduce crystallinity, thereby enhancing the vibrational absorption intensity.^26^

**Fig. 3:**
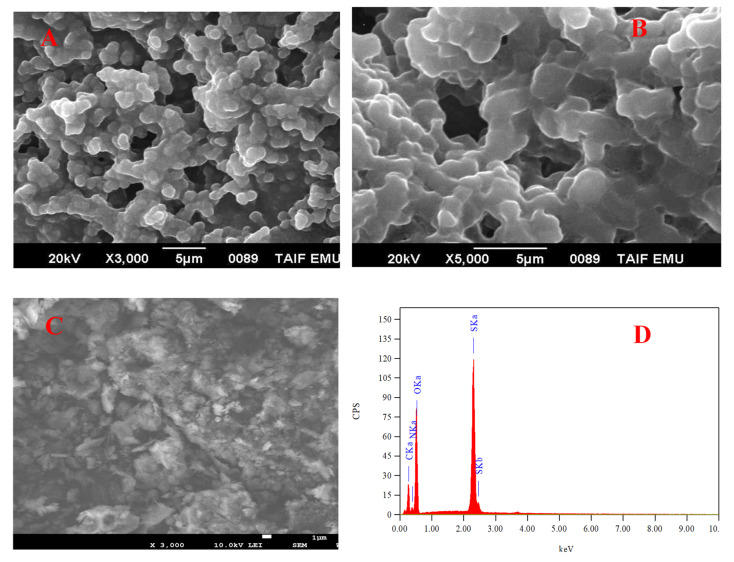
SEM images of SC recorded at different magnification: (A) ×3000, and (B) ×5000, (C) pure chitosan at ×3000, (D) EDX spectrum of SC.

The morphology of SC was investigated using SEM images shown in Fig. 3. The SEM images of SC shows that this material exhibits lower crystallinity compared to pristine chitosan isolated from marine crustacean's waste. The reduction in crystallinity after chitosan sulfonation can be attributed to the introduction of bulky sulfonic acid groups (–SO_3_H) onto the chitosan backbone since native chitosan possesses a relatively ordered semi-crystalline structure stabilized by extensive intra- and intermolecular hydrogen bonding among hydroxyl and amino groups. After sulfonation, the newly introduced sulfonic acid groups disrupt these hydrogen-bonding interactions and increase steric hindrance between adjacent polymer chains. Consequently, the regular packing of chitosan chains becomes less efficient, leading to increased chain spacing, reduced molecular ordering, and a lower degree of crystallinity. This structural disruption promotes the formation of a more open and less compact polymer network. Therefore, the porosity increased as demonstrated in the SEM images taken at the different magnifications. The images recorded at the magnifications of ×300 and ×5000 (Fig. 3A and B, respectively) show a highly interconnected porous structure with pores sizes in submicron to micron range (0.2–1 µm). Such morphology makes SC suitable material to be interlayers since water permeability is expected to increase through membranes containing SC interlayer because of an appropriate porosity. The SEM image of pure chitosan demonstrated in Fig. 3C reveals a dense and compact morphology with a flake-like structure and there is no clear porous network. This indicates strong intra- and intermolecular hydrogen bonding among hydroxyl and amino groups and a relatively high degree of crystallinity. This morphology limits water transport and reduces the availability of active functional groups. On the other hand, use of non-modified chitosan as an interlayer hinders the diffusion of monomers during IP, thereby limiting the formation of a uniform PA layer. Ultimately this reduces water permeability through the membrane.

The EDX analysis of SC shown in Fig. 3D exhibits a distinct sulfur peak, confirming the successful incorporation of sulfonic acid groups into chitosan. Additionally, an increase in oxygen content and a slight decrease in nitrogen content were observed, indicating structural modification. The EDX analysis of pure chitosan (not shown) reveals the presence of C, O, and N elements, with no detectable sulfur signal.

### 3.2. Membranes characterization in terms of chemical structure and morphology

**Fig. 4:**
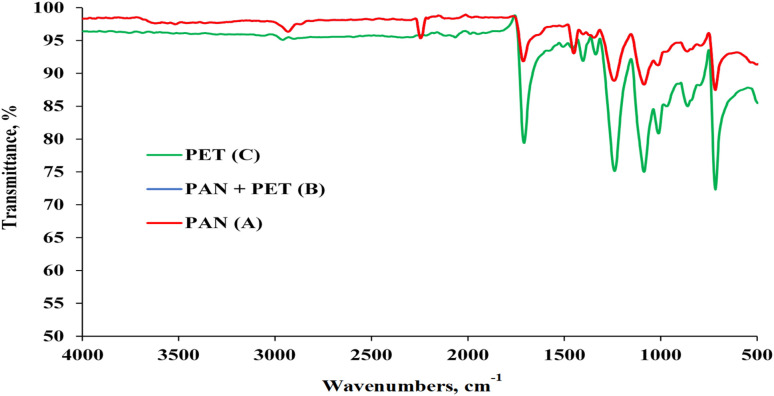
IR spectra of (A) free PAN, (B) PET–PAN substrate, (C) PET.

**Fig. 5:**
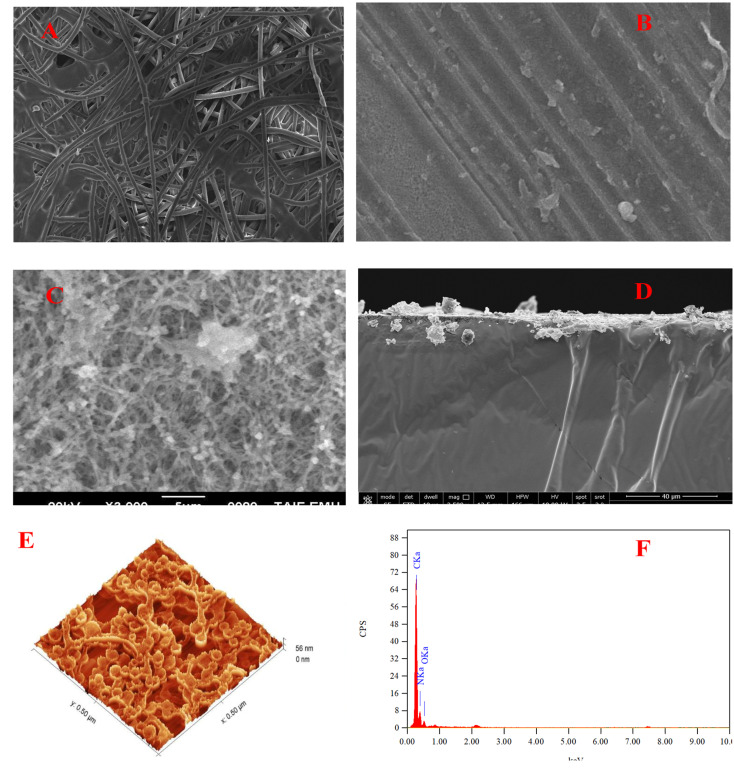
SEM images of (A) free PET, (B) the surface of PAN substrate, (C) PAN surface in the presence of PEG, (D) SEM cross-sectional image of PAN substrate, (E) AFM 3D surface images of PAN substrate, (F) EDX spectrum of PAN substrate.

Firstly, PAN substrate was prepared by the phase inversion technique and characterized using ATR-FTIR and SEM-EDX techniques. To prepare substrate, PAN layer was fixed on PET sheets. Therefore, ATR-FTIR spectra were recorded for free PAN, PET–PAN substrate, and PET as revealed in Fig. 4A–C, respectively. The spectra of free PAN and PET–PAN are absolutely identical, confirming complete coverage of PET surface by PAN where the outermost surface of substrate is PAN layer.^27^ Characteristic bands at 2245 and 1630 cm^−1^ reverting to the stretching frequencies of C≡N and C=N, respectively were observed in the spectra of free PAN and PET–PAN substrate. These bands are the fingerprint of PAN. On the other hand, there are bands of low importance in identifying the structure of PAN substrate including band of C–H stretching at 2850 cm^−1^, and another one at 1050 cm^−1^ for C–C stretching.^27^ ATR-FTIR spectrum of free PET is shown in Fig. 5C for comparison. The ATR-FTIR spectrum of PET is characterized by a strong absorption band in the spectral range of 1715–1730 cm^−1^ which serves as its primary spectral signature. This strong band is attributed to the ester carbonyl (C=O) stretching vibration. On the other hand, there are prominent bands at 1240–1260 cm^−1^ and 1090–1120 cm^−1^ reverting to asymmetric and symmetric C–O–C stretching of the ester bond, respectively. Additionally, there are weak additional bands including aromatic C=C stretching vibrations at 1400–1500 cm^−1^ and out-of-plane C–H bending of the benzene ring in the spectral range of 725–875 cm^−1^, along with aliphatic C–H stretching bands at 2850–2950 cm^−1^.

The SEM images reveal a clear morphological transformation of PET before and after fixing PAN. The free PET surface (Fig. 5A) exhibits a highly porous, randomly entangled fibrous network with significant inter-fiber voids. Such morphology indicates a rough and open structure which facilitates fluid transport. After PAN deposition (Fig. 5B), the surface became markedly more compact and homogeneous. Therefore, the fibrous features observed on free PET surface were fully covered by a PAN layer, leading to a noticeable reduction in pore visibility and overall surface porosity. On the other hand, some traces of separate PAN aggregates were observed on the substrate surface. These aggregates were most probably formed in the PAN solution during its preparation and then attached to the thin PAN film fixed on PET. Such aggregates were previously observed in many papers even with continuous stirring of the casting solution.^27^ As a conclusion, PAN immobilization transforms the PET surface from a porous fibrous morphology into a denser and more uniform structure, which is expected to reduce effective pore size. Although this morphology promotes selectivity, the water permeability will be significantly reduced. However, the surface of PET–PAN substrate has dramatically improved after the addition of PEG-1000 as an additive in the PAN solution. The concentration of PEG-1000 was tested in the range of 1–7 wt%. In fact, the presence of PEG-1000 as a pore-forming agent with the concentration of 3 wt% made the PET–PAN substrate more porous with a little number of aggregates as shown in Fig. 5C. The substrate surface after addition of PEG-1000 is characterized by the formation of uniformly distributed nano-sized pores and nodular features. The pores sizes were measured using Image J software to fall within the range of 50–200 nm. Although the concentrations above 3 wt% provided good results in terms of porosity and water flux, the membrane selectivity with respect to solute rejection lowered. Moreover, adding 3 wt% of PEG-1000 improved the degree of cross-linking as shown in Fig. 5C. Therefore, this concentration was added to PAN solution when preparation of PAN substrate in subsequent work. Thus, PET–PAN–PEG will be named PAN substrate in the next text. The SEM cross-sectional image of PAN substrate shows a characteristic structure with vertical oriented finger-shaped channels (Fig. 5D). Such structure is typical for membranes with high-performance filtration where such channels provide effective water-transport pathways.^28^ Based on the SEM cross-sectional image, the estimated width of finger-like channels in the PAN substrate is in the range of 2–6 µm, with narrower neck regions close to the top surface. On the other hand, the compact top surface supports interlayer deposition and the subsequent formation of PA layer. Moreover, the presence of micrometer-sized channels in the PAN substrate minimizes internal mass transfer resistance, and therefore a high hydraulic pressure is not required to get elevated water flux. Here, the operating pressure is predominantly governed by the resistance of the outer PA layer and osmotic effects instead of the porous substrate structure.^29^Fig. 5E shows 3D AFM topography of the PAN substrate. 3D AFM image with a scan size 0.5 × 0.5 µm reveals a heterogeneous surface with relative roughness at the nanoscale (height variation of 56 nm). Actually, the surface roughness of PAN substrate provides enough topographic characteristics to promote mechanical interlocking with sulfonated chitosan layer. However, the overall adhesion between two layer is strengthened by intermolecular interactions *e.g.* hydrogen bonding and electrostatic interaction between the functional groups of PAN and SC. Based on AFM image, the roughness characteristics *i.e.* root-mean-square roughness (*R*_q_) and average surface roughness (*R*_a_) were calculated to be 21.6 and 18 nm, respectively. The elemental compositions of PAN substrate studied by EDX were displayed in Fig. 5F and Table 1.

**Table 1:** The composition of the membranes and O/N ratio

Membrane	C	N	S	O	O/N (%)
PAN	57.97	34.16	—	7.89	23.09
PAN–SC	52.63	34.86	0.06	12.45	35.71
PAN– SC–PA	56.98	36.8	0.05	6.84	18.5
PAN–PA	58.5	36.4	—	5.01	13.7

**Fig. 6:**
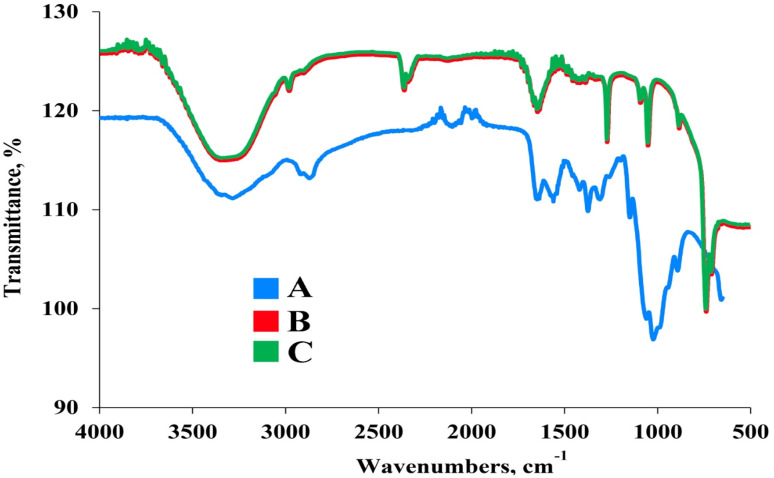
The IR spectra of the developed membranes (A) PAN–SC, (B) PAN–SC–PA, (C) PAN–PA.

ATR-FTIR spectrum of PAN–SC demonstrates the presence of distinct bands at 1034 and 1192 cm^−1^ corresponding to symmetrical and asymmetric vibrations of O=S=O group, respectively in SC.^30^ In fact, the spectrum of PAN–SC is very similar to that of free sulfonated chitosan discussed previously in detail, where the outer layer in PAN–SC is SC material. The strong similarity between the two spectra provides clear evidence of the complete coverage of the PAN substrate surface by the SC layer.^27^Fig. 6B and C show FT-IR spectra of developed membrane (PAN–SC–PA) and control one (PAN–PA), respectively. As revealed in Fig. 6B and C, these spectra are identical since the external surface of two membranes consists solely of PA layer. The distinct stretching frequencies in these spectra are observed at 3230 and 1641 cm^−1^ which revert to N–H and C=O groups (in amide I), respectively. The band of amide II is observed at 1550 cm^−1^ corresponding to N–H bending.

**Fig. 7:**
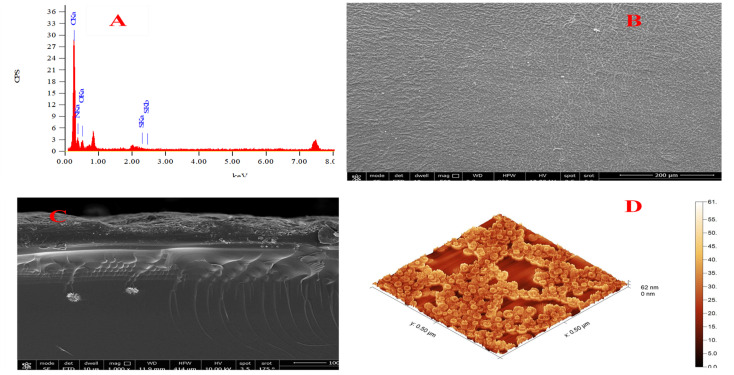
(A) EDX analysis of PAN–SC membrane, and the morphology of PAN–SC membrane: (B) SEM image of membrane surface, (C) SEM image of a cross-section, (D) AFM 3D surface image.

The successful deposition of SC was confirmed by EDX analysis to study elemental composition (Fig. 7A). The EDX spectrum reveals clear peaks of carbon, nitrogen, and oxygen, with distinct sulfur signal at 2.3 keV. In-depth analysis of EDX spectra allows us to compare the elemental content in the prepared membranes. The content of oxygen, as shown in Table 1, increased from 7.89 of substrate to 12.45 of a membrane containing SC as an interlayer (PAN–SC). This is because of OH and SO_3_H present in SC. On the other hand, the oxygen to nitrogen ratio of PAN–SC was 35.71. However, after IP, this ratio decreased to 18.5 for PAN–SC–PA membrane. This is most likely credited to the increase of N content because of the formation of complete layer of PA by IP. In fact, the SC interlayer enhanced the adsorption of an aqueous piperazine solution on the substrate; therefore, a high amount of this substance will react with TMC to produce a denser, and complete layer of PA.^27,31^

After the deposition of SC, the surface of PAN substrate dramatically changed where the SEM surface image of the PAN–SC membrane demonstrated in Fig. 7B reveals a more uniform and continuous morphology compared to PAN substrate. On the other hand, the surface pores and irregular features observed in the original PAN are completely covered by the deposited SC layer. This confirms successful formation of SC interlayer on PAN surface. The resulting surface has a relatively smoother and more homogeneous structure with fine granular features. Therefore, it is expected that IP will be enhanced by the homogeneous distribution of the PIP solution on the surface of PAN–SC membrane yielding a more homogeneous and defect-free PA layer. The SEM cross-sectional image of the PAN–SC displayed in Fig. 7C shows the deposition of a continuous interlayer of SC on the PAN surface. This layer partially penetrates into the upper region of the porous structure without blocking the underlying finger-like channels as shown in Fig. 7C. Thus, the SC deposition did not compromise the internal porosity. The average thickness of SC interlayer was estimated by the SEM cross-sectional images to be approximately 100 nm. This thickness is sufficient for preserving the underlying porous structure of the PAN substrate, thus, maintaining high water permeability.^32^ The AFM image of the PAN–SC membrane displayed in Fig. 7D reveals a distinct nodular surface with well-distributed nanoscale aggregates. Compared to the PAN substrate, the roughness of PAN–SC surface noticeably increased, as evidenced by maximum height variation (∼62 nm) with *R*_a_ = 30 and *R*_q_ = 36 nm. However, the roughness was uniformly distributed, indicating the formation of a homogeneous SC layer. Such morphology enhances surface area and promotes IP.^33^

**Fig. 8:**
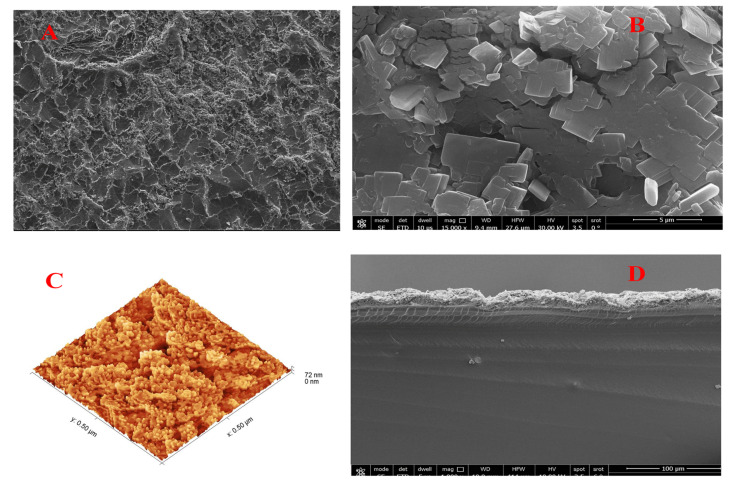
The morphology of PAN–SC–PA membrane, (A and B) SEM images of membrane surface at different magnifications, (C) AFM 3D surface images, (D) SEM cross-sectional image.

**Fig. 9:**
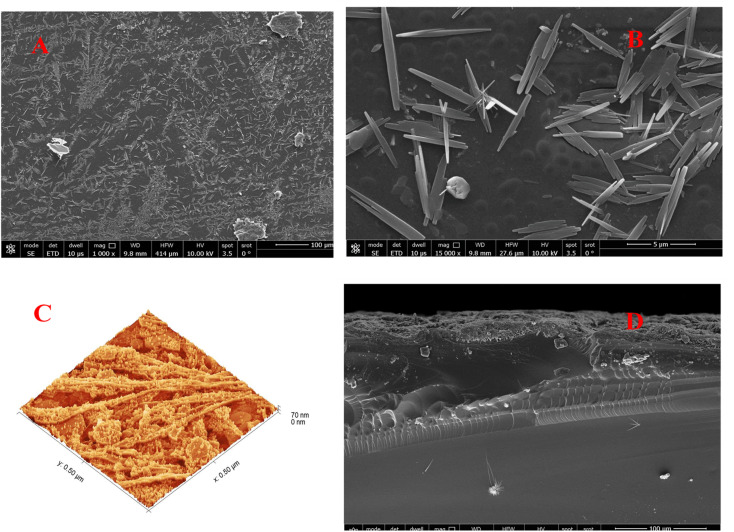
The morphology of PAN–PA membrane, (A and B) SEM images of membrane surface at different magnification, (C) AFM 3D surface images, (D) SEM cross-sectional image.

The outer PA layer in the developed PAN–SC–PA has a continuous surface with moderate heterogeneity and clear roughness as revealed in Fig. 8A. This type of relative heterogeneity is very common in membranes prepared by IP. Such morphology observed on PAN–SC–PA surface may increase the effective surface area, enhancing water permeability.^34,35^ This conclusion was supported by measuring BET surface area of PAN–SC–PA to be 170.64 m^2^ g^−1^ which is larger than that of PAN–SC (137.261 m^2^ g^−1^). SEM image taken at high magnification (Fig. 8B) demonstrates relatively rough surface dominated by distinct plates-like microstructures with small interstitial voids between these microstructures. These voids are localized and non-continuous. Therefore, they did no form impressive defects on PAN–SC–PA surface. On the contrary, the presence of these voids can facilities the formation of additional transport pathways which explains why the PAN–SC–PA membrane provided the significant enhancement of water permeability as it is noted in the subsequent discussion.^36^ Furthermore, SEM image of PAN–SC–PA surface recorded at low magnification (Fig. 8A) does not demonstrate wide cracks, or visible peeling areas indicating the absence of impressive defects. The AFM 3D image of PAN–SC–PA demonstrated in Fig. 8C reveals a morphology with a dense and well-developed nodular surface of PA layer formed by IP. The surface exhibits nanoscale roughness that is relatively higher than those of PAN substrate and PAN–SC where the height variation is approximately 72 nm, whereas, *R*_a_ and *R*_q_ are 40 and 48 nm, respectively. The formation of continuous and defect-free PA layer is suggested by the uniform distribution of nodular features. Despite the structural heterogeneity in outer layer of PAN–SC–PA membrane, this membrane provided an improved rejection efficiency of sodium sulfate as will be discussed later. This performance may be attributed to the presence of SC interlayer which enhances surface hydrophilicity and charge density. The SEM cross-sectional image of compact membrane (PAN–SC–PA) shown in Fig. 8D clearly reveals a thin-film composite structure consisting of porous substrate, thin interlayer, and dense top layer. Moreover, this image reveals good interfacial adhesion between layers of PAN, SC, and PA, indicating the improvement of structural integrity of membrane. The top PA layer appears as a rough and continuous layer without visible defects. The thickness of this layer was estimated to be 286 nm that is less than the thickness of same layer in PAN–PA membrane which was 350 nm in average. It should be noted that the hydrogen bonding forming between the active functional groups *i.e.* found in SC and the groups of N–H in piperazine will reduce the thickness of the outer layer (PA).^25^Fig. 9 shows the morphology of PAN–PA for comparison. SEM images of PAN–PA demonstrated in Fig. 9A and B show a poorly organized morphology with heterogenous surface. Uncontrolled IP was supported by SEM image taken at low magnification (Fig. 9A) where this image shows that the membrane surface appears non-uniform with irregularly distributed domains and visible discontinuities. SEM image captured at high magnification (Fig. 9B) reveals isolated needle-like structures with the presence of large interstitial voids. These voids are different from those observed on PAN–SC–PA surface where the current voids are irregular, relatively large, and interconnected. Such voids may create non-selective transport pathways, influencing the selectivity of membrane. AFM analysis of PA layer in PAN–PA reveals relatively low roughness with the height variation of 63 nm and an irregular surface as demonstrated in Fig. 9C. This topography indicates uncontrolled IP and growth of a non-uniform film. Although the PAN–SC–PA has slightly high roughness (72 nm) compared to control membrane (62 nm), the roughness nature is fundamentally different. The control membrane shows irregular and defect-prone roughness, whereas, PAN–SC–PA membrane presents a more uniform and well-defined nodular structure. As previously mentioned, the controlled roughness as in the case of PAN–SC–PA membrane is useful as it can enhance effective surface area and improve water transport pathways with maintaining structural integrity and high salt rejection.^37^ The cross-sectional SEM image of control membrane shown in Fig. 9D clearly demonstrates irregular and less continuous PA layer with interfacial discontinuities and formation of large voids. On the other hand, Fig. 9D reveals that the thickness of outer layer varies from one area to another along the membrane because of the irregularity of this layer. The irregular morphology suggests a higher probability of nanoscale defects and localized structural weaknesses.^37^ These factors may adversely influence selectivity and overall separation performance of membrane.^38^

### 3.3. Surface features of fabricated membranes

**Fig. 10:**
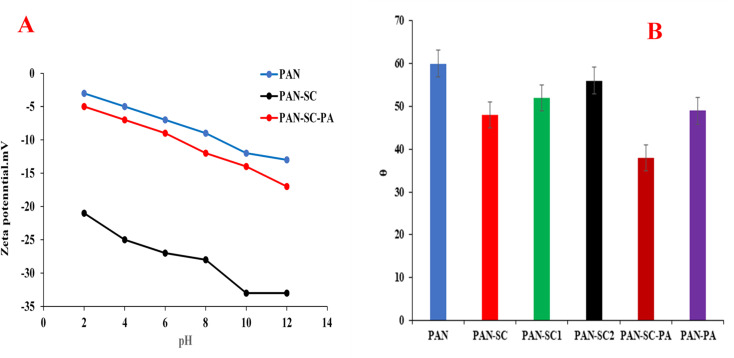
The zeta potential (A), and WCA (B) of prepared membranes.

The zeta potentials of fabricated membranes surfaces, *i.e.* PAN substrate, PAN–SC, and PAN–SC–PA are displayed in Fig. 10A. All membranes under study were negatively charged under a wide range of pH (2–12). However, the PAN–SC is the most negatively charged membrane because of sulfonic acid groups (–SO_3_H) in SC where these groups are deprotonated in a wide range of pH. The surface of developed PAN–SC–PA membrane containing SC as an interlayer is more negative compared to PAN substrate because of the presence of sulfonated chitosan carrying negative charges. On the other hand, the control membrane, PAN–PA, (not shown) has less negative charge than PAN–SC–PA. Therefore, the presence of sulfonated chitosan significantly enhanced the negative charge on the surface of PAN–SC–PA membrane. Thus, the Donnan exclusion effect is enhanced by strengthening the electrostatic repulsion towards multivalent anions such as sulfate ions.^39^ This enhancement in the negative surface charge of PAN–SC–PA membrane could explain its superior Na_2_SO_4_ rejection performance.

The water contact angle (WCA) is a measure of how a water droplet spreads on a surface. When WCA is smaller, this means better wetting (more hydrophilic), whereas, larger WCA means poorer wetting (more hydrophobic). Typically, a hydrophilic membrane has WCA <90°, while, the same angle is larger than 90° for a hydrophobic one. Fig. 10B shows WCA for PAN substrate, PAN–SC, and PAN–SC–PA membranes surfaces. Generally, there is a correlation between hydrophilicity and surface roughness of membranes. According to Wenzel model, the increase in surface roughness enhances the wettability of hydrophilic surfaces by increasing solid–liquid contact area. Therefore, the WCA values reduces with increasing surface roughness. The previous discussion of membranes surfaces morphology reveals that the order of roughness was PAN–SC–PA > PAN–SC > PAN substrate. Therefore, WCA of these membranes is in reverse order with surfaces roughness as shown in Fig. 10B. The WCA of PAN–SC membranes deceases when increasing the deposition time of SC where the WCA of PAN–SC (deposition time of 5 h) is smaller than those of PAN–SC1 and PAN–SC2 with deposition time of 3 h and 2 h, respectively. The decrease in WCA with increasing SC deposition time can be attributed to increasing SC amount deposited on PAN surface over the time, enhancing the surface hydrophilicity. The improved hydrophilicity of PAN–SC enhances IP due to the regular distribution of the aqueous PIP solution on the surface of this membrane reducing the defects in PA layer.^40^ Moreover, the presence of SC interlayer made PAN–SC–PA membrane more hydrophilic than PAN–PA where WCA of the control membrane (PAN–PA) was larger compared to that of PAN–SC–PA as shown in Fig. 10B. Therefore, PAN–SC–PA membrane will have high effective water permeance.

### 3.4. The relation between preparation conditions and membrane efficiency

**Fig. 11:**
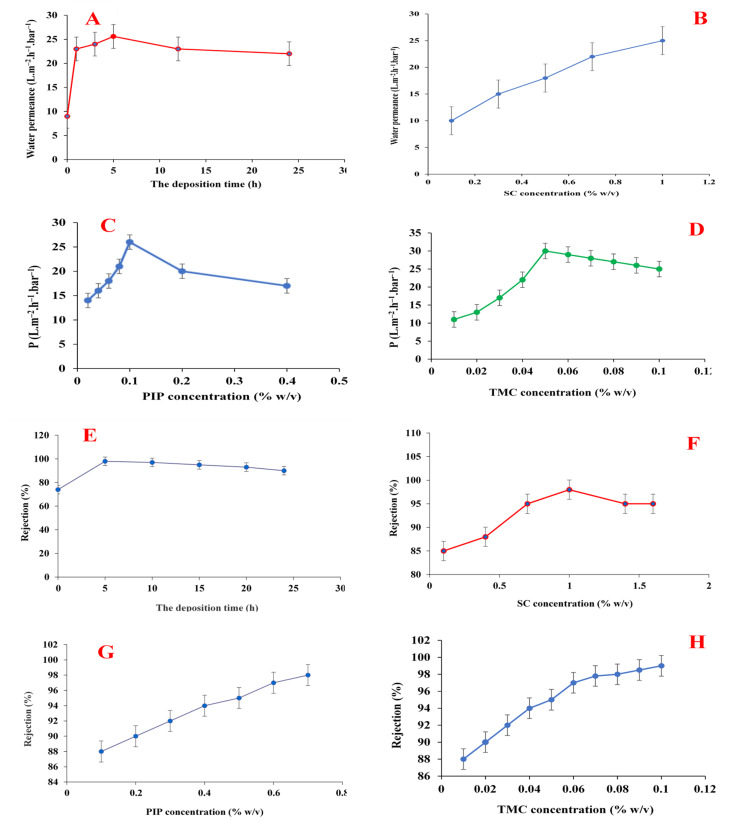
The influence of preparation conditions on PAN–SC–PA membrane efficiency water permeance: A–D; Na_2_SO_4_ rejection: E–H.

In general, the optimized PAN–SC–PA membrane provided a high performance in terms of water permeance (25.6 L m^−2^ h^−1^ bar^−1^) and salt rejection (99.02% for Na_2_SO_4_) compared to control PAN–PA membrane. It can be said that the presence of SC interlayer provided short pathways for water molecules through PA layer, reducing of total permeation resistance.^37^ This conclusion is consistent with the morphological study of the external PA layer in the PAN–SC–PA membrane previously discussed. However, the performance of PAN–SC–PA is strongly affected by manufacturing conditions as shown in Fig. 11. It should be noted that the deposition time of SC has a strong effect on the water permeance of PAN–SC–PA membrane where increasing this time reduces the permeance as shown in Fig. 11A. This may lead to the conclusion that prolonged deposition time may cause pores blockage of the PAN substrate as a result of excessive interlayer accumulation on its surface. Fig. 11E shows that excessive SC deposition time let to a clear reduce in Na_2_SO_4_ rejection. The reduce in Na_2_SO_4_ rejection can be attributed to main two reasons, (i) the formation of an overly thick interlayer, which interferes with IP process. This leads to the development of a less uniform PA selective layer creating defects or loosely crosslinked regions which facilitate the transport of salt ions through the membrane. (ii) Excessive accumulation of SC can reduce the effective exposure of negatively charged SO_3_H groups on the PAN–SC–PA surface. This phenomenon weakens the Donnan exclusion effect toward sulfate ions and consequently decreasing salt rejection efficiency.^4,5^ However, at the deposition time of 5 h, PAN–SC–PA membrane demonstrated a high water permeance of 25.6 L m^−2^ h^−1^ bar^−1^ and an excellent Na_2_SO_4_ rejection of 99.02%%. Therefore, this time is selected in subsequent work.

Not only the deposition time of SC influences the water permeance of the PAN–SC–PA membrane, but also its concentration in the deposition solution plays a significant role in determining membrane performance, where water permeance improved from 10.2 to 25.6 L m^−2^ h^−1^ bar^−1^ with changing SC concentration from 0.12 to 1% w/v as revealed in Fig. 11B. This is due to the increase of SC amount deposited on PAN surface with increasing SC concentration. Thus, the increase of SC concentration in deposition solution enhances water permeance because of the higher density of hydrophilic functional groups *e.g.* –SO_3_H that improve membrane hydration and facilitate water transport through selective PA layer.^11^ Anyway, the increase above 1.00 wt% caused a reduce in the rejection ratio of Na_2_SO_4_ to 95.9% as demonstrated in Fig. 11F with slight increase in water permeance. In fact, some defects in PA layer were observed with increasing SC concentration. One of these defects is non-uniform PA layer thickness observing as ridge–valley structure or wavy surface morphology under SEM. Thus, we used the concentration of 1.00 wt% in next work because this concentration provided the best balance between water permeability and Na_2_SO_4_ rejection.

The concentrations of trimesoyl chloride and piperazine should be optimized to obtain an excellent performance of polyamide membranes. The effect of piperazine concentration was examined in a wide range of 0.01–0.4 wt%. Fig. 11C shows almost linear increase in *P* of PAN–SC–PA membrane as the PIP concentration increased. However, the increase above 0.1 wt% caused a clear decrease in water permeance. It is well known that the high amount of PIP can increase the density of amine groups available to react with TMC which often leading to thinner or more crosslinked PA layer enhancing water transport. At high concentrations (>0.5 wt%), uneven polymerization may happen leading to some surface defects and excessive roughness in PA layer. Here, a clear reduce in water permeance with enhancement of the rejection of Na_2_SO_4_ were observed as revealed in Fig. 11C and G. The impact of TMC concentration on the desalination performance of PAN–SC–PA membrane was studied in the range 0.01–0.1 wt%. Fig. 11D shows a clear increase in the water permeance in the range (0.01–0.05 wt%). However, the Na_2_SO_4_ rejection reduced to 90% (Fig. 11H) with increasing TMC concentration in the same range. Although *P* reduced from 30 to 25 L m^−2^ h^−1^ bar^−1^ when increasing concentration in the range of 0.05–0.1% w/v, the Na_2_SO_4_ rejection was achieved the maximum (99.02%) at 0.1 wt%. The main reason for this behavior is probably due to an increase of cross-linking degree in outer layer of PAN–SC–PA membrane (PA layer).^38,40^ From the previous discussion, the influence of concentrations of both PIP and TMC on the membrane efficiency is opposite. PIP and TMC concentrations of 0.1 and 0.05 wt%, respectively offered the best balance of solute rejection and water permeance. Thus, these concentrations were used for the fabrication of PAN–SC–PA membranes.

### 3.5. Desalination performance of PAN–SC–PA membrane

After optimization of membranes fabrication conditions, desalination performance of developed PAN–SC–PA membrane was studied in details.

#### 3.5.1. Filtration performance of developed PAN–SC–PA membrane

**Fig. 12:**
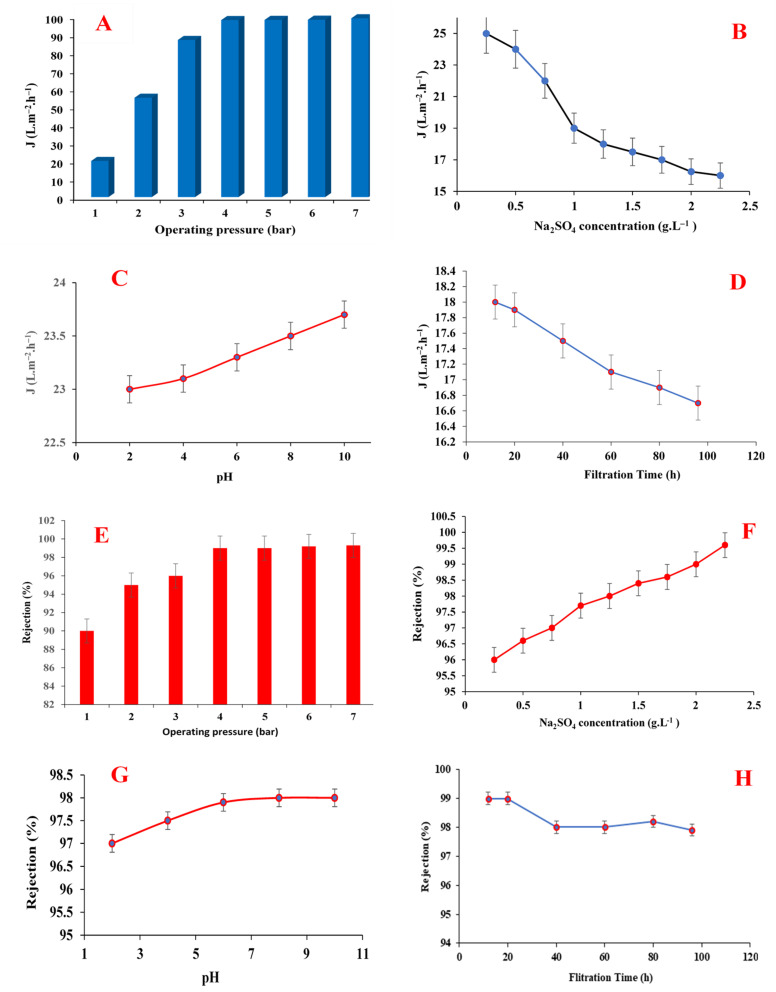
Filtration efficiency of PAN–SC–PA membrane in terms of water flux (A–D) and Na_2_SO_4_ rejection (E–H).

In membrane processes driven by pressure, the water flux is directly proportional to the effective operating pressure, assuming other factors remain constant. Therefore, the influence of pressure on the performance of developed PAN–SC–PA membrane was studied from 1 to 7 bar. As exposed by Fig. 12A, the pure water flux increased linearly from 20 at 1 bar to 98 L m^−2^ h^−1^ at 4 bars. At pressures above 4, the pure water flux remains constant. Spiegler–Kedem model (Eq.(5)) explains this behavior where the effect of osmotic pressure is very low at low concentrations, it can therefore be neglected.^41^ While, the rejection of Na_2_SO_4_ increases gradually (Fig. 12E) because salt flux is almost constant according to Spiegler–Kedem model. The rejection of Na_2_SO_4_ achieved to 99% at 4 bars. Thus, the pressure of 4 bars was taken as an operating pressure in the next experiments.

The performance of PAN–SC–PA membrane significantly changed when saline solutions are employed instead of pure water where osmotic pressure, ion–membrane interactions, transport phenomena, and concentration polarization are dominant factors governing water transport and salt rejection. Here, it is better to use the term of water flux rather than water permeance. Fig. 12B shows that water flux decreased gradually in Na_2_SO_4_ concentration range of 0.25 to 2.25 g L^−1^ at the pressure of 4 bars, whereas, the rejection of Na_2_SO_4_ increased as shown in Fig. 12F. This behavior is expected according to Spiegler–Kedem model. This model tells us that osmotic pressure will increase as the Na_2_SO_4_ concentration in the feed solution increases. Therefore, the water flux will reduce according to eqn (5):

5 *J* = *L*_p_(Δ*P − σ*Δπ)

where, *L*_p_ is hydraulic permeance of membrane, Δ*P* is the difference of applied pressure through membrane, Δπ is the change of osmotic pressure, *σ* represent reflection coefficient. The rejection of sodium sulfate increased with increasing feed concentration, which can be attributed to the strong electrostatic repulsion between the negatively charged membrane surface and divalent sulfate ions, together with the large hydration radius and low diffusivity of sulfate species within the PA selective layer.^5^

The impact of feed solution pH on the effectiveness of compact PAN–SC–PA membrane was investigated in a wide range from 2 to 10. When studying the impact of feed solution pH on the effectiveness of PAN–SC–PA, it should be remembered that the PAN–SC–PA membrane surface has negative charge at a wide range of pH values where sulfonic acid groups (–SO_3_H) in SC is ionized at pH 1–10.^42^ Therefore, it is expected that the rejection of negative ions such as sulfate ion by PAN–SC–PA membrane does not affect dramatically with changing feed solution pH. Fig. 12G confirms this expectation where the rejection of Na_2_SO_4_ increased rapidly at the beginning, then slight changes were observed with increasing pH values. Moreover, Fig. 10A shows that the increase of the negative charge on the PAN–SC–PA surface is slight with increasing pH. On the other hand, Fig. 12C illustrations that the water flux slightly increased with increasing feed solution pH. This behavior provides further evidence that increasing feed solution pH does not significantly affect PAN–SC–PA surface charge. From the previous discussion, increasing pH slightly enhanced both water flux and salt rejection. The slight dependence of water flux and salt rejection on feed solution pH is most likely attributed to the stable ionization of sulfonic acid groups over a wide pH range, maintaining constant negative surface charge. The broad-range ionization behavior of sulfonic acid (–SO_3_H) groups ensures sustained membrane hydrophilicity and a stable negative surface charge enhancing water permeance and preserving effective Donnan exclusion. Therefore, desalination performance using the PAN–SC–PA membrane will be stable over a wide pH range. Na_2_SO_4_ solution of 2.25 g L^−1^ was employed to study the stability of developed membrane through filtration process. This solution was filtrated at a pressure of 4 bar for 96 h. Fig. 12D and H show the water flux and the rejection ratio of sodium sulfate, respectively. Although both water flux and the rejection ratio of sodium sulfate slowly lowered over time, the rejection of Na_2_SO_4_ (97.9%) and water flux (16.7 L m^−2^ h^−1^) are still high even with 96 h of filtering. The superior long-term stability observed for the PAN–SC–PA membrane can be attributed to the multifunctional role of the SC interlayer. First, the hydrophilic sulfonic groups facilitate the formation of a strongly bound water layer, which alleviates flux decline during prolonged operation. Second, the SC interlayer serves as an intermediate bridging layer between PAN and PA, this reduces interfacial stress and enhances structural integrity. Therefore, the negligible variation in water permeance and salt rejection over 96 h confirms that the SC interlayer not only enhances separation performance but also significantly improves membrane durability and resistance to operational decay. Although no specific foulants were introduced during the stability test, the improved hydrophilicity imparted by the SC interlayer is expected to reduce foulant adsorption and contribute positively to fouling resistance in practical applications. Table 2 show a comparison between the efficiency of polyamide membranes in the absence (PAN–PA) and in the presence (PAN–SC–PA) of SC interlayer. In fact, the presence of SC interlayer improved the water permeance dramatically (25.6 ± 0.5 L m^−2^ h^−1^ bar^−1^). This means that the PAN–SC–PA membrane exhibited a dramatic increase in water permeance of approximately 1064% compared to the PAN–PA. The Na_2_SO_4_ rejection improved from 95% for PAN–PA to 99.02% for compact membrane. Despite a modest relative increase (∼4.5%), the rejection improvement from 95% to 99.3% represents a substantial enhancement in desalination efficiency. The simultaneous enhancement in water permeability and salt rejection can be attributed to a synergistic effect of increased surface hydrophilicity, strengthened Donnan exclusion due to sulfonated functional groups, and the formation of more efficient water transport pathways within the modified polyamide layer.^43^

**Table 2:** Comparison the efficiency of polyamide membranes in the absence (PAN–PA) and in the presence (PAN–SC–PA) of SC interlayer^a^

Comparison element	PAN–PA	PAN–SC–PA
The permeance of pure water (L m^−2^ h^−1^ bar^−1^)	2.2 ± 0.3	25.6 ± 0.5
The rejection of Na_2_SO_4_ (%)	95.3 ± 0.6	99.02 ± 0.2

a Operation conditions: Na_2_SO_4_ concentration, 2.25 g L^−1^; operation pressure = 4 bar.

#### 3.5.2. Salts rejection behavior and separation mechanism of PAN–SC–PA membrane

**Fig. 13:**
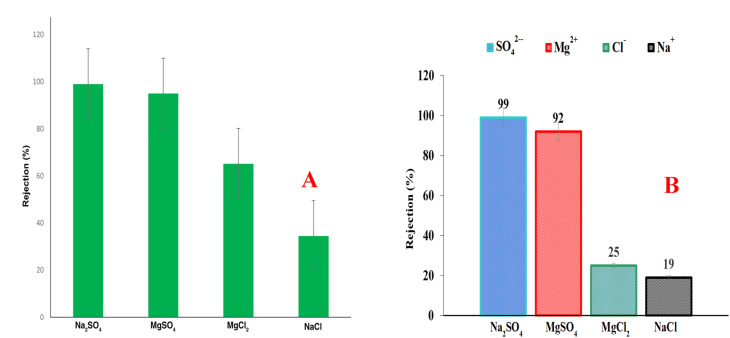
The separation efficiency of PAN–SC–PA membrane, the sequence of salts rejection (A), and the sequence of ions rejection (B).

Set of salts were employed to test the efficiency of PAN–SC–PA, and Fig. 13A shows the results. The highest rejection efficiency was for Na_2_SO_4_ (99.02%), whereas, the lowest was for NaCl (34.5%). The sequence of salts rejection shown in Fig. 13A indicates that the Donnan effect based upon the electrostatic repulsion is dominant. It is well-known that the electrostatic repulsion of monovalent anions is weaker than that of divalent anions. Therefore, the rejection of chloride ion was less than sulfate ion. On the other hand, the PAN–SC–PA membrane rejected Na_2_SO_4_ with higher ratio than MgSO_4_ (95%). This phenomenon may be described by the influence of charge shielding. It should be noted that positive charge density of Mg(ii) ion, divalent cation, is higher than that of Na^+^ ion (monovalent cation). Therefore, Mg^2+^ ion can lower the efficiency of PAN–SC–PA membrane towards anions by shielding the negative charges found on surface of this membrane. Consequently, the rejection of sodium sulfate is higher than that of magnesium sulfate. It is assumed that PAN–SC–PA membrane will reject MgCl_2_ with low rejection ratio compared to NaCl according to charge shielding effect. However, the rejection ratio of sodium chloride (34.5%) was less than that of magnesium chloride (65.2%). Here, the main role in this order is the influence of steric hindrance. Smaller Na^+^ ions (hydrated radius = 0.358 nm) will pass more easily through the membrane compared to larger Mg^2+^ ions (hydrated radius = 0.428 nm). On the other hand, it is possible to say that the interaction between Mg(ii) ions and –SO_3_^−^ groups present in SC interlayer may play a crucial role in enhancing MgCl_2_ rejection. The partial binding of Mg(ii) ions increases its retention time, and combined with electroneutrality constraints, creates an electrostatic bottleneck that hinders ion transport. This effect, coupled with the strong Donnan exclusion, leads to a significantly higher rejection of divalent salts compared to monovalent ones.^44^ Based on the above discussion, the observed salt rejection sequence can be summarized by the synergistic effects of steric and electrostatic interactions. Sodium sulfate exhibited the highest rejection owing to the combined influence of Donnan exclusion and low charge-shielding effects associated with sodium ions. Conversely, sodium chloride showed the lowest rejection rate due to its relatively small hydrated ions, which experience weaker steric hindrance and a less significant electrostatic exclusion effect. Magnesium salts displayed intermediate rejection behavior, exceeding that of NaCl but remaining below that of Na_2_SO_4_, primarily due to the synergistic contribution of steric hindrance and the pronounced charge-shielding effect of Mg^2+^ ions.

The selectivity of PAN–SC–PA membrane was studied by using a NaCl/Na_2_SO_4_ mixture as a feed solution. The rejection of both SO_4_^2−^ and Cl^−^ were 99% and 30.9%, respectively at filtration time of 60 min and operation pressure of 4 bars. Here, the selectivity of developed PAN–SC–PA membrane 
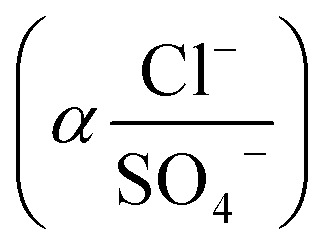
 was 77.9. Thus, PAN–SC–PA membrane separated these ions each other effectively. Chloride ions have less charge density than sulfate ions, while, sulfate ion has radius larger than that of chloride. Stokes radius of sulfate ion and chloride ion are 0.230 nm and 0.121 nm, respectively. Therefore, Donnan effect and sieving effect are dominant on separation mechanism. Fig. 13B shows the efficiency of PAN–SC–PA membrane towards a mixture containing four species of inorganic salts. The rejection sequence of ions by the PAN–SC–PA membrane followed the order SO_4_^2−^ > Mg^2+^ > Cl^−^ > Na^+^. This behavior can be attributed to the combined effects of electrostatic repulsion (Donnan exclusion) and steric hindrance. The membrane surface possesses a high negative charge density due to the presence of ionized sulfonic acid groups (–SO_3_^−^) within the SC interlayer. Consequently, sulfate ions (SO_4_^2−^), carrying a double negative charge, experience the strongest electrostatic repulsion, resulting in the highest rejection. Magnesium ions (Mg^2+^) exhibit higher rejection than monovalent ions because of their larger hydrated radius and stronger hydration shell, that increase steric resistance during transport through the membrane. In contrast, chloride ions (Cl^−^) are subjected to weaker electrostatic repulsion than sulfate ions due to their lower charge valence, leading to moderate rejection. Sodium ions (Na^+^) demonstrate the lowest rejection because they are electrostatically attracted to the negatively charged membrane surface and possess a relatively small hydrated size, facilitating their passage through the membrane.

**Fig. 14:**
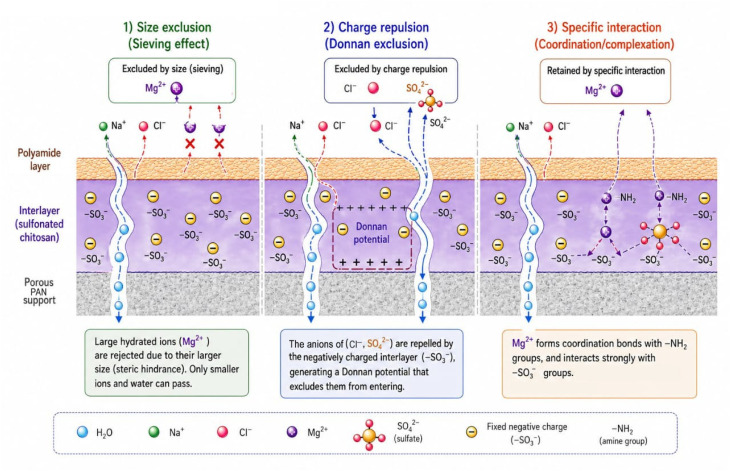
A schematic illustration of the potential separation mechanisms.

From previous discussion, the interlayer in PAN–SC–PA membrane is highly electronegative and hydrophilic. Hence, the presence of this interlayer makes PAN–SC–PA membrane surface has high electronegativity. This may explain why the rejection rate of cations is less than that of anions with the same valence regardless of the size. On the other hand, PAN–SC–PA membrane can reject the divalent ions (anions or cations) effectively. Here, the electrostatic repulsion represents the most important mechanism in the rejection of divalent anions, while, the pore size screening is controlled in divalent cations rejection. The separation of monovalent ions from divalent ions was efficiently attained by steric effect where most of monovalent ions can pass through membrane easily due to their small radius. A schematic illustration of the potential separation mechanisms of PAN–SC–PA membrane developed in the present work is shown in Fig. 14.

#### 3.5.3. Comparison of membrane performance

We compared the efficiency of developed membrane in the current study with some commercial membranes and those stated in the literature.^43–52^ As revealed in Table 3, the PAN–SC–PA membrane provided a superior desalination performance compared with some previously reported membranes, achieving simultaneously high water permeance (25.6 L m^−2^ h^−1^ bar^−1^) and excellent Na_2_SO_4_ rejection (99.02%). These results clearly demonstrate the ability of the SC interlayer to overcome the conventional trade-off effect between permeability and salts rejection, which remains one of the major challenges in PA membranes engineering. As previously discussed, the improved performance of PAN–SC–PA membrane is attributed to the synergistic role of the SC interlayer in enhancing membrane hydrophilicity, and stabilizing negative surface charge, and therefore promoting the formation of a thin yet highly selective polyamide layer.

**Table 3:** Comparison of the performance efficiency of developed membrane with other membranes^a^

Membranes	Operating pressure (bar)	Water permeance (L m^−2^ h^−1^ bar^−1^)	Rejection (%)		Separation factor αNaCl/Na_2_SO_4_	pH	Ref.
			Na_2_SO_4_	NaCl			
PAN-PCA_2_-PA	6	102.8	99.1	30.2	75.9	6.0	43
CHDA-TMCPES	10	8.27	99.1	N.D.	80	9.4	44
Z-CNF@TFN	4	14.4	98.3	12.4	9.2	6.5	45
TA/Fe-TFC	5.7	19.6	95.6	15.8	18.9	4	46
TFN/UiO-66-NH_2_	6	13.0	98.1	19.0	42.6	8.5	47
TFN-PDP	6	9.85	98.0	44.1	59.1	11.2	48
PA/MoS_2_/ceramic	4	17.7	96.8	<40	N.D.	3.6	49
PA/PDA-COF/PAN	2	20.7	93.4	19.0	N.D.	6.0	50
PSf/GO-vanillin	5	10.4	92.5	25.4	N.D.	7.5	51
NF90	10	7.00	99.1	89.6	N.D.	9	52
PAN–SC–PA	4	25.6	99.02	34.5	77.9	2–8	This study

a PAN: polyacrylonitrile; PCA_2_; poly(caffeic acid); PA: polyamide; CHDA: *trans*-1,4-diaminocyclohexane; TMC: trimesoylchloride; MCPES: polyethersulfone; Z-CNF: zwitterionic cellulose nanofiber; TFN: thin-film nanocomposite; TA: tannic acid; TFC: thin-film composite; PDP: polydopamine-piperazine; NF: nanofiltration; PDA: polydopamine; COF: covalent organic framework; PAN: polyacrylonitrile; SC: sulfonated chitosan; N.D.: not determined.

## 4. Conclusion

This work addresses the trade-off effect by introducing a SC interlayer derived from marine wastes. The methodology presented in the present work modulates monomer diffusion during IP, enhances surface hydrophilicity and charge density, and therefore, achieves synergistic improvement in water permeance and salt rejection. Chitosan isolated from shrimp was converted to sulfonated chitosan by chlorosulfonic acid, then this material was used as an interlayer to manufacture PAN–SC–PA membrane. The phase inversion method was used to prepare PAN substrate, while, dead-end ultrafiltration was applied to deposit SC interlayer on the surface of this substrate. The successful deposition of interlayer was confirmed by IR spectrum, SEM images, and the analysis of EDX results. The fabrication of PA layer on the surface of PAN–SC membrane was carried out by IP. Different techniques were used for comprehensive structural and morphological studies of prepared membranes. The morphological studies and surface properties of SC revealed that this material is more suitable than chitosan itself as an interlayer for manufacturing filtration membranes due to many features such as high hydrophilicity, the low degree of crystallinity and high selectivity of anions. Therefore, a composite polyamide membrane prepared in this work provided good desalination performance where *P* and *R* of Na_2_SO_4_ were 25.6 L m^−2^ h^−1^ bar^−1^, and 99.02%, respectively. The selectivity of composite membrane 
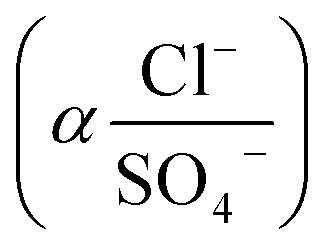
 was 77.9. Moreover, PAN–SC–PA membrane gave high stability during 96 h of continuous filtration.

## Conflicts of interest

The authors declare that they have no known competing financial interests or personal relationships that could have appeared to influence.

## Data Availability

Data supporting this study are openly available from the corresponding authors
